# Influence of Laser Energy Density on Tribological Properties of AlSi10Mg Manufactured by Selective Laser Melting

**DOI:** 10.3390/ma17020323

**Published:** 2024-01-09

**Authors:** Keqing Wang

**Affiliations:** School of Automation, Wuxi University, Wuxi 214105, China; 860020@cwxu.edu.cn

**Keywords:** tribological properties, AlSi10Mg, SLM, laser energy density, regression model

## Abstract

In recent years, much work related to the performance of AlSi10Mg manufactured by selective laser melting (SLM) has been extensively researched. However, the study of tribological performance caused by different laser energy densities is still insufficient. This work concentrates on the relationship between the wear resistance and laser energy density of AlSi10Mg processed using SLM. Moreover, XRD characterization, density, surface roughness and microhardness were also examined since they are closely related to wear resistance. The results revealed that the XRD pattern of AlSi10Mg was mainly composed of the α-Al and Si phases under the conditions of different laser energy densities. In addition, the peak of Mg_2_Si was also detected. Also, the grain size increased with the increasing of laser energy density. The increase in laser energy density led to an increase in the convection and porous phenomenon in the molten pool. However, when the value was lower, the overlapping area reduced, and the strength between adjacent melting paths was insufficient, resulting in the declination of the sample property. According to the experimental results, a laser energy density of 63.33 J/mm^3^ was considered to be a relative optimal condition. The relative density, Ra, microhardness and wear volume were 99.2%, 8.86 μm, 128.3 HV_0.2_ and 2.96 × 10^−2^ mm^3^, respectively. The worn surface morphology also confirmed the influence of laser energy density on wear resistance. A regression model was established and analyzed, which showed the reliability of the results. Furthermore, the tribological mechanism was also revealed.

## 1. Introduction

AlSi10Mg has been massively deployed in the aerospace, automotive and marine industries in recent years due to its high heat conductivity, low weight and outstanding mechanical properties [1,2,3]. However, the traditional forging or casting process still has limitations due to its time-consuming nature and low cost-effectiveness [4,5,6]. In the wake of technology developments, selective laser melting (SLM) gradually became a hot topic owing to its high free-forming and rapid prototyping ability [7,8]. Due to the advantages of SLM, researchers began to focus their attentions on it, and a thorough study was also conducted into improving the quality of SLM-formed parts.

It is also worth noting that tribological property is extremely important in practice since it is considered as one of the most important factors in determining the service life of the printed part, and it has been reported in recent research [9,10,11,12]. Mishra et al. [13] investigated the anti-wear performance of SLM-manufactured AlSi10Mg with different scan strategies. Steel ball bearings were used as a standard sliding counterpart material to investigate the wear properties and obtained optimum wear rate and friction coefficient with an island scan strategy—3.76 × 10^−6^ mm^3^/Nm and 0.0781, respectively. Gao et al. [14] prepared AlSi10Mg with SLM and researched the influence of scan speed on tribological properties. Experimental results indicated that the microstructure was remarkably refined with a gradually increased scan speed, and wear resistance improved accordingly. Xi et al. [15] manufactured AlSi10Mg with SLM at different laser powers and found the friction coefficient decreased with the increase in laser power; a similar trend also occurred in the wear rate. A 3 mm diameter G-Cr15 steel ball bearing with an average hardness of HRC60 was used against the composite materials. When the laser power was 450 W, the friction coefficient was 0.65 and the corresponding minimum wear rate was 5.2 × 10 mm^3^/Nm. Wu et al. [16] surveyed the relationship between the sliding speed and anti-wear property of AlSi10Mg fabricated via SLM and discovered the decreasing of wear rate with the increasing of sliding speed. The counterbody material was GCr15 with a hardness of HRC63 ± 3 and with a diameter of 4 mm. Meanwhile, the coefficient of friction also decreased under the same variation range.

From the above analysis, it can be concluded that the influence of SLM parameters on the tribological properties of AlSi10Mg has been studied but not thoroughly. It was reported that more than 130 process parameters had an impact on the performance of SLM-formed parts, of which about 13 parameters were crucial [17]. Among these, laser power and scan speed were considered as key parameters, while hatch spacing and layer thickness, which are closely related to laser energy density, were also worth considering [18]. Hong et al. [19] systematically investigated the impact on the performance of AlSi10Mg formed via SLM at different laser energy densities, which consisted of pore formation, microstructure and mechanical properties. The results showed that different laser energy densities was the main reason for the above performance changes. Chen et al. [20] formed AlSi10Mg and analyzed the mechanical properties, while obtaining the best overall performance when the laser energy density was 44.5 J/mm^3^. Maamoun et al. [21,22] explored the influence of laser energy density on the properties of SLM-manufactured AlSi10Mg and demonstrated that microstructure and mechanical properties can be controlled via selecting the SLM process parameters. In addition, it was found that the optimal values of different properties corresponded to different laser energy densities. For example, when the hardness was optimal, the laser energy density was 65 J/mm^3,^ while when the ultimate tensile strength and density were optimal, the corresponding values were 60 J/mm^3^ and 55 J/mm^3^, respectively.

Considering all the above mentioned, it can be conclusively found that the effect of laser energy density on the microstructure of AlSi10Mg has been studied, but few studies reported the influence of laser energy density on tribological property. As reported in the relevant research [10,15], laser energy density has a great impact on grain size and quality, which further affects the tribological property of the printed parts. So, in this work, the role of laser energy density on microstructure, density, surface roughness (Ra) and micro-hardness were also explored, given their close relationship to wear resistance. Residual stress of the samples was not measured in this work.

## 2. Experimental Section

### 2.1. Materials

**Figure 1:**
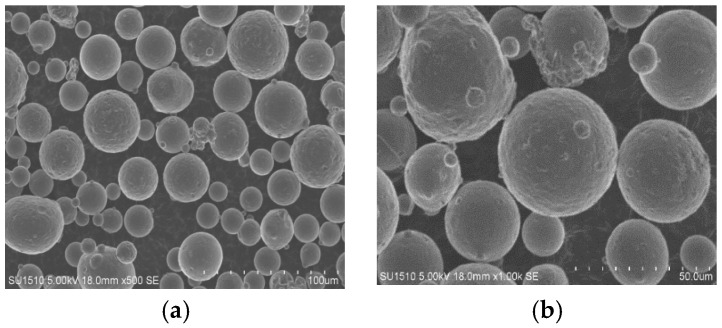
Morphology of AlSi10Mg powder shown in (**a**,**b**).

AlSi10Mg powder with 15–53 μm diameter particle size was manufactured using gas atomization method and provided by Avimetal Powder Metallurgy Technology Co., Ltd., Beijing, China. Particle size, element content and morphology are shown in Table 1 and Figure 1.

### 2.2. SLM Process Parameters

The SLM instrument was YLM-120 provided by Jiangsu Yongnian Laser Co., Ltd., Kunshan, China. Single mode fiber laser was used in this instrument with the smallest spot size of 30 µm. Argon was inserted in the chamber to prevent oxidation in printing process. To prevent error in the experiments, three samples were printed under the same process parameters. The size of the specimen was 10 mm × 10 mm × 10 mm. Laser power, scan speed and hatch spacing are listed in Table 2. Laser energy density was calculated as follows [21,22].

(1) $$E=\frac{P}{V\cdot D\cdot H}$$

where *E*, *P*, *V*, *D* in this equation represented laser energy density (J/mm^3^), laser power (W), scan speed (mm/s), hatch spacing (mm), while layer thickness (*H*) was kept at a constant value of 25 µm.

### 2.3. Measurement Methods

X-ray diffractometer (XRD), Bruker D8, Ettlingen, Germany, was used to test the phase composition at a rate of 2.0°/min with LIF and RSD. Microhardness was tested using HVS-1000ZCM- XYY instrument provided by Shanghai Suoyan, Shanghai, China. In the testing process, the pressure was kept at 200 N for 12 s and the authors chose 5 places for testing (four corners and the most central point). Vickers hardness was employed in this work. Optical microscope (OM) DM-2700M, Leica, Wetzlar, Germany, was used to observe the optical morphology. Before testing, each specimen was polished with abrasive paper. The density was determined using Archimedes drainage method and calculated with following formula [23].

(2) $$\varphi =(1-\frac{M}{\left(V^{\prime }-V\right)\rho })\times 100\%$$

In this equation, φ, *M*, ρ, $V^{\prime }$ and V represented the relative density, weight, part density, theoretical density, the volume of water before and after of the sample, respectively.

Wear resistance was tested using reciprocating friction and wear-testing machine, MFT-5000, RTEC, San Jose, CA, USA. The normal pressure was 10 N while the frequency was 1 Hz. A 304 stainless steel ball with a diameter of 3.262 mm was applied as counter grinding ball. The hardness and roughness value of the counter ball were HRC 26 and Ra 5 μm, respectively The displacement and test time were set to 10 mm and 0.5 h, respectively. The friction coefficient curve and wear volume were exported and calculated directly by the built-in software.

Morphology was scanned by white light interferometer, MFP-D, RTEC, USA. In addition, worn surface was also investigated through scanning electron microscope (SEM), Carl Zeiss, Sigma 300, Rödermark, Germany. For the sake of results’ accuracy, all specimens were tested three times and the mean value was calculated. It should be noted that although the properties of all samples were tested, only representative data were analyzed, that is, the minimum value (31.67 J/mm^3^, S18) and maximum value (101.33 J/mm^3^, S7) of laser energy density, and values corresponding to better wear resistance performance (60.00 J/mm^3^, S5 and 63.33 J/mm^3^, S14).

## 3. Results and Discussion

### 3.1. XRD Characterization

**Figure 2:**
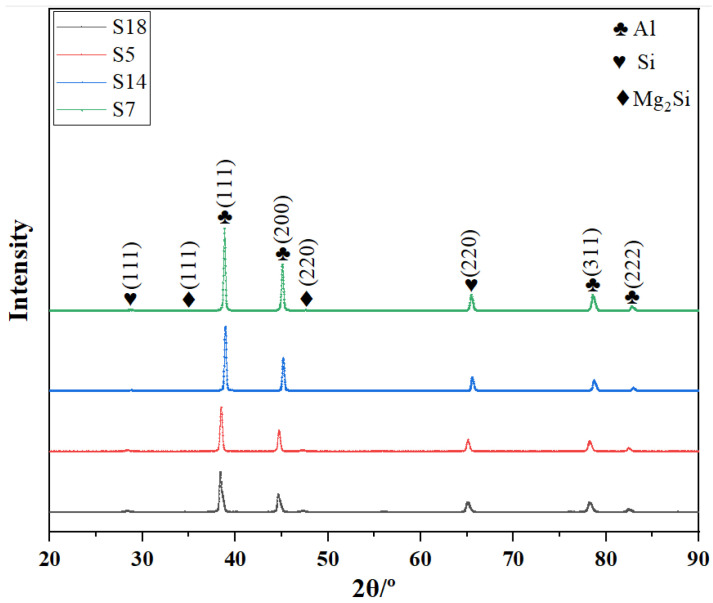
XRD of AlSi10Mg samples formed at different laser energy densities.

For SLM-formed AlSi10Mg, XRD characterization patterns for different laser energy densities are shown in Figure 2. This figure indicates that the phase composition of AlSi10Mg formed via SLM is mainly composed of the α-Al and Si phases. In addition, the diffraction signal of the Mg_2_Si phase was also detected but not obviously, which may be due to the lack of Mg content and Mg_2_Si phase [24,25,26]. The average FWHM of Al peaks was recorded on the basis of different XRD, and the grain size changes were also calculated according to Scherrers’s equation [21]:

(3) $$D=\frac{k\lambda }{\beta cos\theta }$$

In this equation, *D* represented grain size (nm), k was Scherrer’s constant (0.9), λ indicated wavelength of X-ray (0.15406 nm), β was FWHM, while θ was peak location.

The average FWHM and grain size of AlSi10Mg specimens at different laser energy densities are shown in Table 3. It can be seen that the average grain size increased with the increasing of laser energy density, which indicated that low laser energy density was more likely to lead to wider FWHM. In addition, studies have shown that the sharpness of peaks in XRD patterns was positively correlated with the grain size [22]. From Figure 2, it can be seen that the peak in the XRD spectrum became sharp gradually with laser energy density increasing, which also confirmed the change process of grain size from fine to coarse. At low laser energy density, the overall temperature of the molten pool was low and the solidification time was short, which led to a relatively better grain size. On the contrary, when higher laser energy density acted on the metal powder, it absorbed more energy, and the overall temperature of the molten pool was also higher. The molten pool with a higher temperature needed more solidification time, resulting in longer nucleation growth time, which leads to a larger grain size at higher laser energy density.

### 3.2. Density and Surface Roughness (Ra)

**Figure 3:**
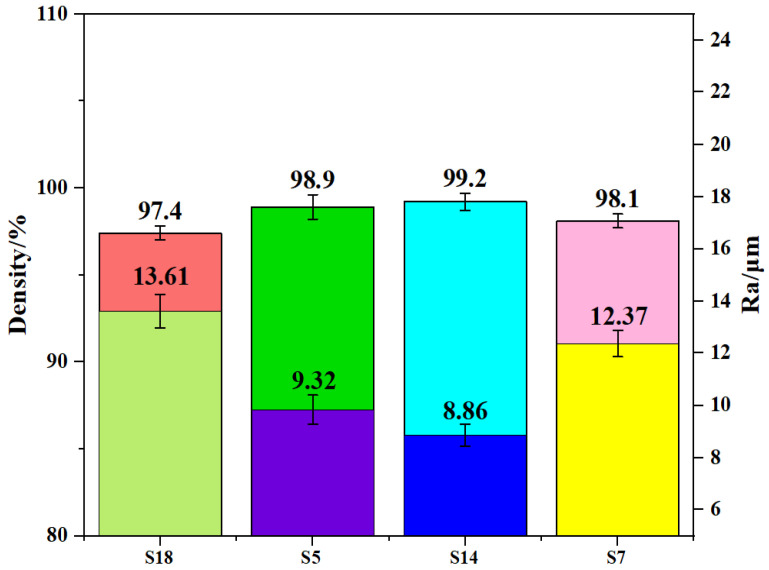
Density and Ra of AlSi10Mg samples.

As shown in Figure 3, it can be distinctly seen that the density of all AlSi10Mg samples is higher than 97.4%. With the increase of laser energy density, the density first increases and then decreases. By the time it arrives at 60.00 J/mm^3^ (S5), the density is 98.9%, showing it increased to a certain extent. As the laser energy density continues to increase, the density improves to 99.2%, which is relatively optimal in the results gained in this work, but the increment is not obvious. Most obviously, the density does, however, decrease from 99.2% to 98.1% along with laser energy density’s further increase. From the viewpoint of surface topography, the varying trend is opposite to the density. That is, high density corresponds to low surface roughness (Ra).

The reason why the value of density was quite small when laser energy density is 31.67 J/mm^3^ (S18) or 101.33 J/mm^3^ (S7) can be explained by the porous formation in the SLM process. With the increasing of laser energy density, the Ra appeared to show a downward trend. Studies have shown that, when laser energy density was too high, the convective heat transfer presented an unstable trend [22,23,24,25,26]. This caused the increase in the non-uniformity of the molten pool temperature distribution, leading to powder splashing in the overlapping area during the SLM process. Powder splashing occurring in the forming process not only affected the quality of the printing layer, but also caused the unevenness of the layer, resulting in the overall performance degradation of the samples. Apart from this reason, when laser energy density was too small, the strength between each scan line also decreased. Moreover, when the distance between each scan line increased, the molten pool was unlikely to flow to the overlapping zone before its solidification. It caused the formation of large voids in the overlapping zone and decreased the density of the printed part. If it is too low, a mass of unmelted powder may be generated inside the specimen, the molten pool cannot form an effective lap and obvious gullies can be seen on the surface. Too big or small laser power density would disorder the convection in the molten pool, making it difficult for the internal gas to overflow and forming pores which reduce the density of the sample. The change in density can also be explained by the above reasons; higher density and better surface roughness always corresponded to each other. Therefore, the laser energy density was better controlled within a reasonable range.

**Figure 4:**
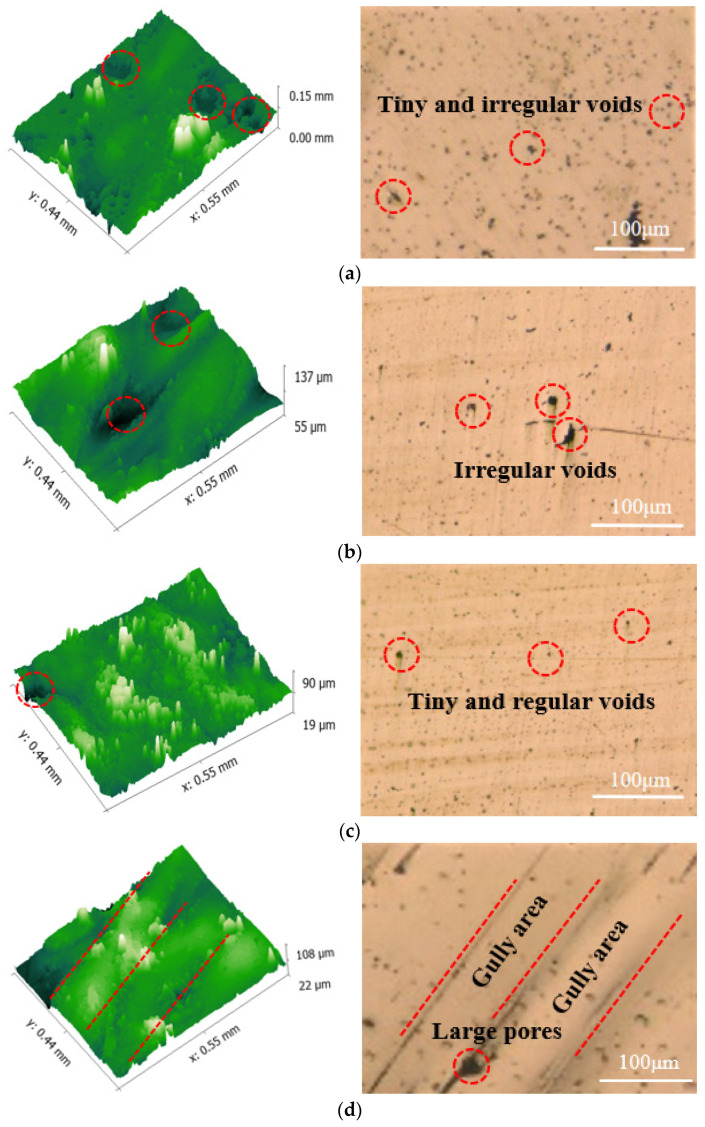
The surface morphology of specimens: (**a**) S18, (**b**) S5, (**c**) S14 and (**d**) S7.

To further verify the explanation given above, surface morphologies are presented in Figure 4. Tiny voids, which had a much lower size compared to large pores, can be seen in Figure 4a,d, respectively. Tiny and irregular voids on the printed specimens can be clearly seen when the laser energy density value was 31.67 J/mm^3^ (S18). As it increased, the pores on the surface became smaller. Furthermore, the voids seemed relatively regular and evenly distributed at a value of 63.33 J/mm^3^ (S14), while some quite obvious pores and gully areas could be found on the surface as it continued to increase. As for the voids seen in S5 and S7, large and irregular voids could still been seen on these samples.

### 3.3. Microhardness

**Figure 5:**
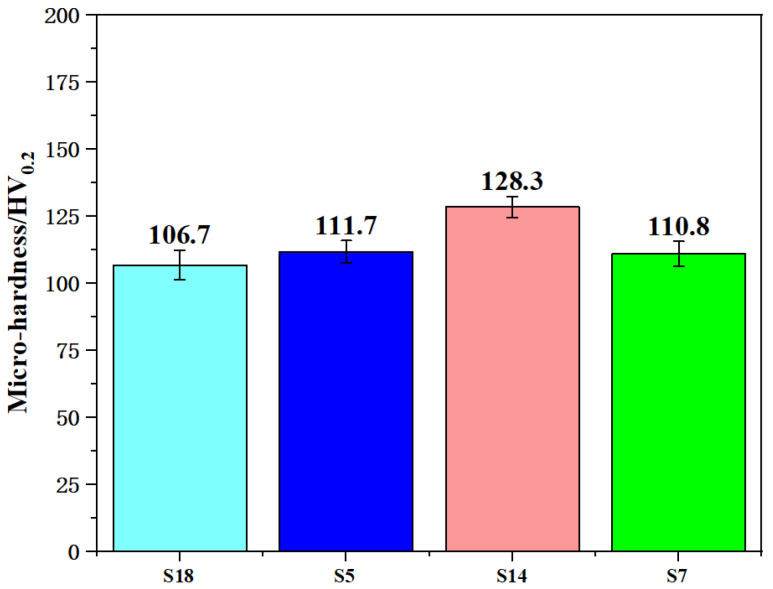
Microhardness of AlSi10Mg specimens formed at different laser energy densities.

The microhardness values can be seen in Figure 5. From the testing results, it can be found that the specimen formed at 63.33 J/mm^3^ (S14) showed a relative optimal micro-hardness value. The phenomenon observed here seemed pretty consistent with that of the density observed before. That is, the overall microhardness increased first and then decreased in pace with the laser energy density increasing, as mentioned above. The microhardness of SLM-formed parts was closely related to density. When laser power, scan speed and hatch spacing were non-fixed values, the forming quality of the single molten pool was basically different. The formation of each layer mainly depended on the overlap between multiple molten pools. When laser energy density was 101.33 J/mm^3^ (S7), the microhardness was 110.8 HV_0.2_. Here, an excessive overlap rate led to a higher molten pool temperature, and the over-melted powder increased the porosity and reduced the microhardness value. In addition, due to the large amount of heat input per unit area, the molten pool temperature of the current processing layer increased rapidly. However, the molten pool of the previous processing layer had fully cooled, resulting in a large temperature gradient between adjacent processing layers, which was not conducive to the combination between the front and rear layers. When it decreased to 63.33 J/mm^3^ (S14), the microhardness increased by 15.8% and reached a relative optimal condition. This was mainly due to the decreasing of the overlap ratio of the molten pool, which reduced the over-melting phenomenon and increased the microhardness. As it continued to decrease, the microhardness slightly decreased. More obviously, when it was 31.67 J/mm^3^ (S18), the microhardness decreased by 20.2% compared to the relative optimal value. This may have been caused by too low laser energy density, which resulted in a lower overlap rate of the molten pool, forming a discontinuous melting path or the inclusion of unmelted powder, which reduced the microhardness value.

### 3.4. Tribological Performance

**Figure 6:**
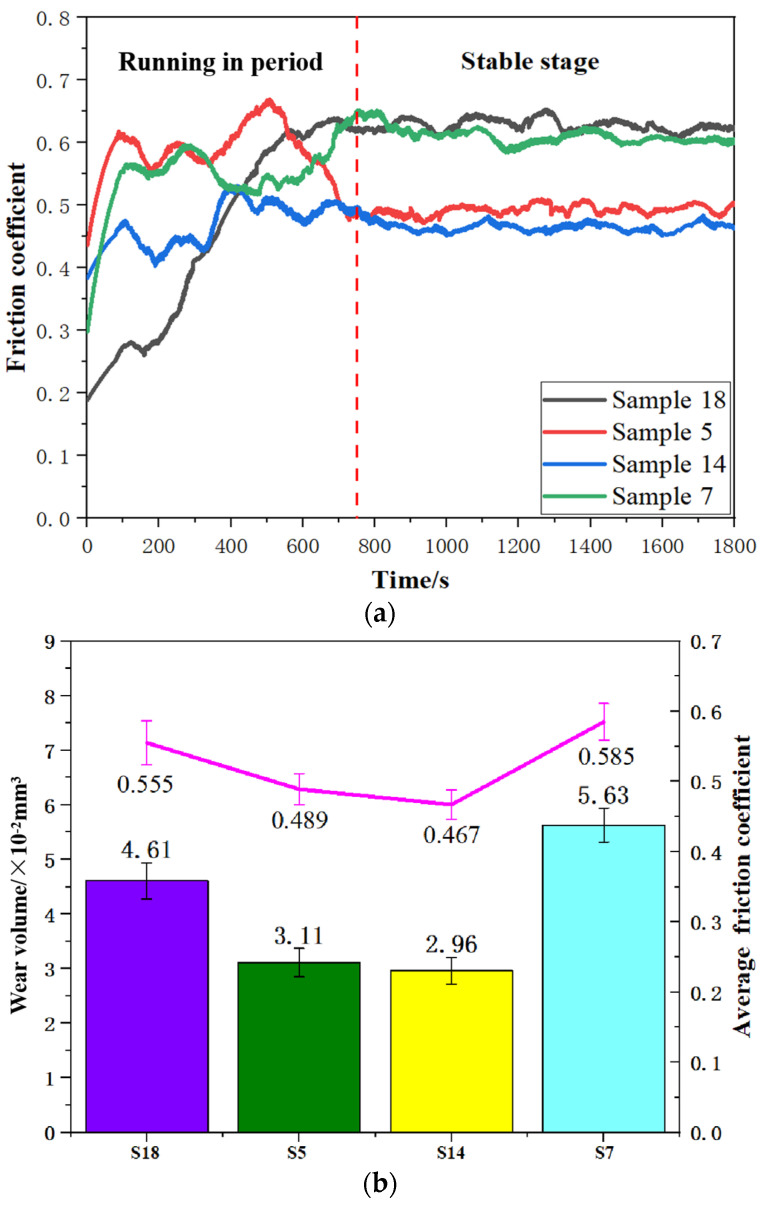
Tribilogocal properties of AlSi10Mg specimens fabricated via SLM: (**a**) friction coefficient, (**b**) average friction coefficient and wear volume.

Figure 6 shows the friction coefficient and wear volume after friction and wear tests. It can be seen in Figure 6a that the initial stage was in an unstable wear period. At this stage, point contact existed between the grinding ball and substrate; the specimen was under great pressure, which intensified the wear and increased the friction coefficient. After 800 s, it reached the stable wear stage. With the increase in contact area, the contact manner between the grinding ball and the specimen turned into surface contact, which decreased the pressure on the specimen. The friction coefficient of Samples 7 and 18 (around 0.6) was significantly higher than that of Samples 5 and 14 (around 0.45) at the stable stage. From Figure 6b, it can be seen that the average friction coefficient fluctuated between 0.45 and 0.60 during the friction process. When laser energy density was 31.67 J/mm^3^ (S18) and 101.33 J/mm^3^ (S7), the friction coefficient was no less than 0.555, which was highly related to the surface morphology of the sample. Too high or too low a value will affect the surface quality of the sample. Many protrusions on the surface were gradually broken under the action of the grinding ball, which led to the abrasive debris’ attachment on the surface. The flaked wear debris was squeezed and bitten to form large wear particles, causing abrasive wear and sharp fluctuations in the friction coefficient. When the laser energy density was 60.00 J/mm^3^ (S5) and 63.33 J/mm^3^ (S14), the friction coefficient decreased by 16.4% and 20.2%, respectively, which was related to the density and microhardness. In a certain range, the density and microhardness were also enhanced with the increasing of laser energy density, resulting in the decreasing of the friction coefficient. The literature also showed that wear resistance was related to microhardness [23,27]. In addition, as shown in Figure 6b, it can be found that the trend of wear volume was similar to the friction coefficient, which was that a low friction coefficient corresponded to a low wear rate [27,28,29,30,31]. When laser energy density was 63.33 J/mm^3^ (S14), the wear volume possessed a minimum value of 2.96 × 10^−2^ mm^3^. Too large or small a laser energy density will increase the wear volume.

**Figure 7:**
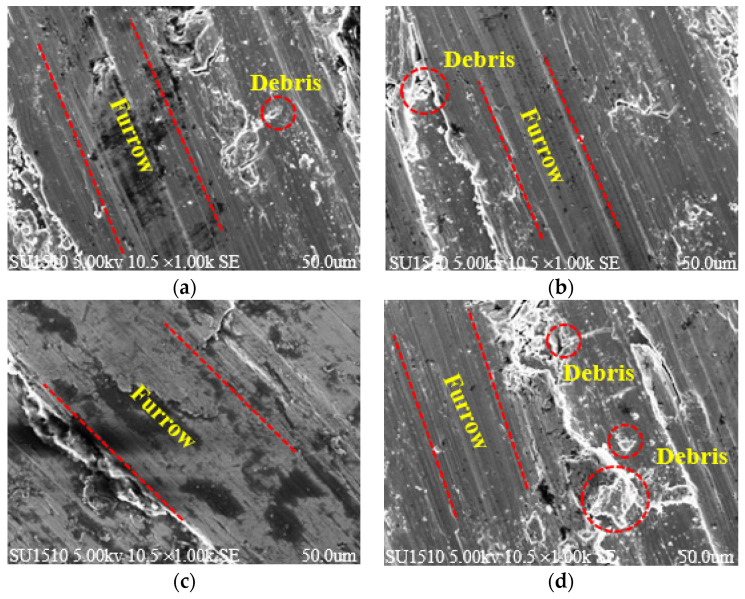
SEM of tribilogocal wear morphology at different laser energy densities: (**a**) S18, (**b**) S5, (**c**) S14 and (**d**) S7.

To further explore the effect of laser energy density on wear resistance, the worn surface morphology was characterized using a SEM, as shown in Figure 7. When the laser energy density was 101.33 J /mm^3^ (S7), a large amount of wear debris and furrows were generated on the wear tracks’ surface due to serious deformation and ploughing in the friction process. With the increasing laser energy density, wear debris and furrows on the wear surface decreased obviously and tended to be flat, which was mainly caused by density and microhardness improvement. However, when laser energy density was 31.67 J/mm^3^ (S18), deep furrows appeared on the wear surface. This is due to the fact that, when it was too small, the discontinuous melting path and unmelted powder caused surface defects which reduced the density and microhardness and led to serious wear. The testing results revealed, that in the dry friction condition, AlSi10Mg formed under the laser energy density value of 63.33 J/mm^3^ (S14) showed relative optimal wear resistance in all these four specimens. It was mainly caused by its relative higher density and microhardness.

**Figure 8:**
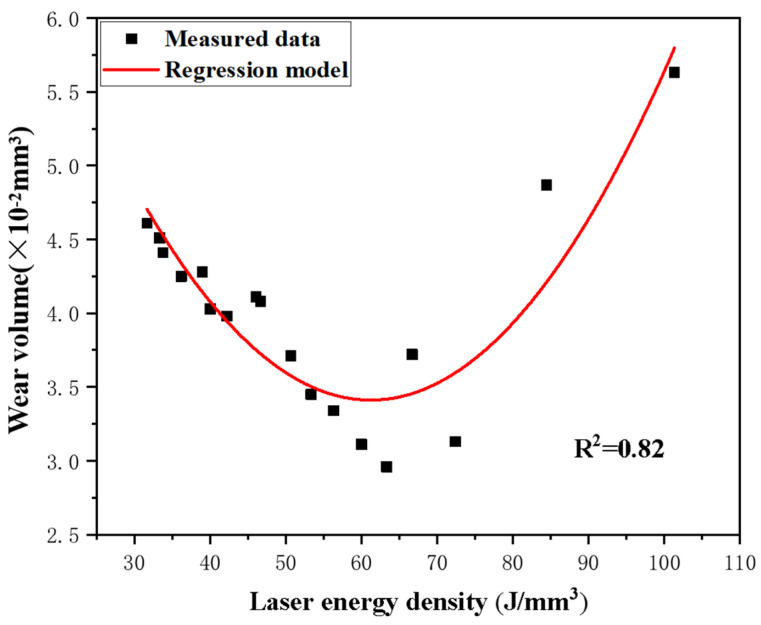
Regression model of laser energy density and wear volume.

As illustrated in Figure 8, the regression model is shown below:

(4) $$Y=0.00148X^{2}-0.18128X+8.95861$$

where *Y* is the predicted wear volume (×10^−2^ mm^3^), while *X* is laser energy density (J/mm^3^).

Figure 8 displays the regression model of laser energy density and wear volume. The results showed that, within the optimal range of SLM process parameters, the AlSi10Mg sample showed a lower wear rate which resulted in better wear resistance. Based on this regression model, the minimum wear volume can be obtained as 3.407 × 10^−2^ mm^3^ at a laser energy density of 61.24 J/mm^3^. According to the actual parameters of this paper and Figure 8, it can be seen that, when the laser energy density varied between 55 and 65 J/mm^3^, the lowest wear volume value was achieved, as low as 2.96 × 10^−2^ mm^3^. The experimental data was basically consistent with the prediction regression model. And the R^2^ value was 0.82, which indicates the reliability of this regression model.

When the laser energy density was not within the reasonable range, the properties of the sample changed significantly. Beyond this range, the wear volume of AlSi10Mg samples increased remarkably. At a lower laser energy density, less input energy led to the lack of fusion of metal powder, resulting in poor microhardness and wear resistance. In practice, however, the higher laser energy density also did not reach satisfactory results. Excessive energy input led to excessive melting of the metal powder, resulting in defects and tribological properties reduction.

**Figure 9:**
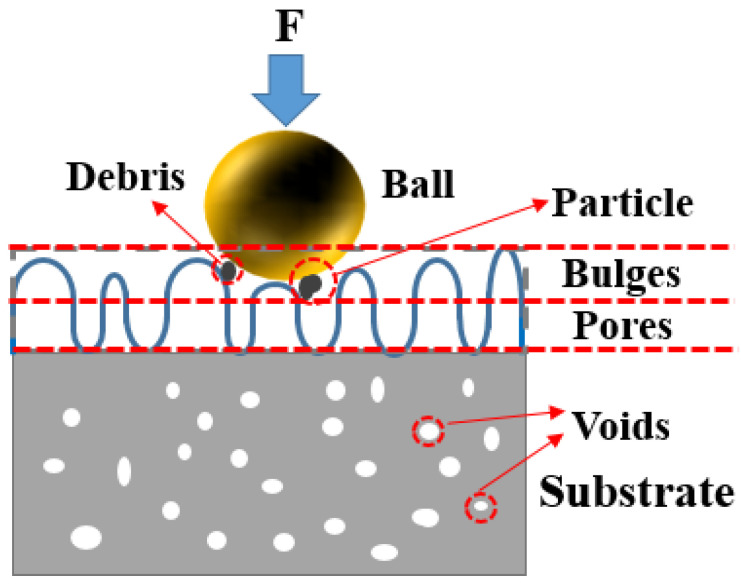
Tribological mechanism of SLM-formed AlSi10Mg.

The tribological mechanism of AlSi10Mg formed via SLM based on different laser energy densities can be seen in Figure 9. Different laser energy densities resulted in different wear resistance. When the laser energy density was too large, bulges and pores were distributed on the sample surface. The bulges were gradually broken down under the action of external force in the process of friction and wear and formed wear debris adhering to the sample surface. The peeling of wear debris formed large wear particles, resulting in abrasive wear and wear resistance reduction. When a small laser energy density was employed, a discontinuous melting path and unmelted powder also affected the wear resistance. If the laser energy density was mismatched, pores could also form in the specimen, which further reduced the microhardness value. When an appropriate value was adopted, the specimen presented better worn surface morphology and microhardness, as well as excellent wear resistance. Another interesting point was the effect of tribo-oxidatives during the test. With the increasing of the sliding distance in the experiment, it generated significant frictional heat that promoted a reaction between Al and O present in the environment, forming aluminum oxide. This debris can restick on the worn surface during the subsequent passages of the pin, promoting the more-or-less continuous formation of an oxide layer and adhering to the tested material. The brittle oxide was progressively broken and removed during the wear test, producing fine debris particles. Given the absence of oxide layers on the wear tracks shown in Figure 7, it can be assumed that the oxidation of the worn surface takes place in the wear test, which may further decrease the COF value in the test due to the contact between the oxidized wear debris adhering to the worn surface of the counterball and the oxide layers formed on the wear tracks.

## 4. Conclusions

In this work, laser energy density was systematically researched to study the impact on the tribological properties of AlSi10Mg manufactured via SLM, including XRD characterization, density, microhardness and wear resistance. Testing results revealed that the wear resistance was affected by laser energy density, and 63.33 J/mm^3^ was considered as a relative optimal value. Detailed conclusions were drawn as follows.

(1) The XRD pattern of AlSi10Mg manufactured through SLM was mainly made up of α-Al and Si phases under different laser laser densities. Moreover, the peak of the Mg_2_Si phase was also detected but not obvious. Also, the grain size grew with the increasing of laser energy density.
(2) The density and microhardness of the specimen first increased and then decreased with the increasing of laser energy density. The maximum value was 99.2% and 128.3 HV_0.2_, respectively. However, the surface roughness (Ra) showed an opposite trend, and the optimal value was 8.86 μm.
(3) The minimum friction coefficient and lowest wear volume of the sample were 0.467 and 2.96 × 10^−2^ mm^3^, and the corresponding laser energy density value was 63.33 J/mm^3^. In this case, the specimen showed a relative optimal wear resistance.

## Figures and Tables

**Table 1:** Particle size and element content of AlSi10Mg powder.

AlSi10Mg	Particle Size/μm			Element Content/wt.%					
	D_10_	D_50_	D_90_	Si	Fe	Mn	Mg	Zn	Al
Value	15.9	30.9	53.7	9–11	0.55	0.45	0.35	0.1	Bal.

**Table 2:** SLM process parameters.

No.	P (W)	V (mm/s)	D (mm)	E (J/mm^3^)	Density (%)	Ra (μm)	Hardness (HV_0.2_)	Friction Coefficient	Wear Volume (×10^−2^ mm^3^)
S1	125	1000	0.15	33.33	97.6	13.43	107.1	0.541	4.51
S2	150	1000	0.15	40.00	97.9	11.43	107.3	0.548	4.03
S3	175	1000	0.15	46.67	98.0	11.28	108.5	0.532	4.08
S4	200	1000	0.15	53.33	98.5	10.04	109.9	0.521	3.45
S5	225	1000	0.15	60.00	98.9	9.84	111.7	0.489	3.11
S6	250	1000	0.15	66.67	98.3	9.32	113.6	0.531	3.72
S7	190	500	0.15	101.33	98.1	12.37	110.8	0.585	5.63
S8	190	700	0.15	72.38	98.5	12.03	120.6	0.510	3.13
S9	190	900	0.15	56.29	98.4	10.15	113.5	0.527	3.34
S10	190	1100	0.15	46.06	98.1	11.58	108.1	0.512	4.11
S11	190	1300	0.15	38.97	98.0	12.35	106.7	0.533	4.28
S12	190	1500	0.15	33.78	97.5	13.57	106.8	0.550	4.41
S13	190	1000	0.09	84.44	98.6	12.43	108.5	0.573	4.87
S14	190	1000	0.12	63.33	99.2	8.86	128.3	0.467	2.96
S15	190	1000	0.15	50.67	98.3	10.64	109.8	0.542	3.71
S16	190	1000	0.18	42.22	98.1	11.59	107.8	0.546	3.98
S17	190	1000	0.21	36.19	97.5	12.22	107.2	0.550	4.25
S18	190	1000	0.24	31.67	97.4	13.61	106.7	0.555	4.61

**Table 3:** Average FWHM and grain size of AlSi10Mg at different laser energy densities.

Laser Energy Densities (J/mm^3^)	31.67(S18)	60.00(S5)	63.33(S14)	101.33(S7)
FWHM (°)	0.30809	0.26399	0.26411	0.23532
Grain size (nm)	27.93	32.58	32.59	36.57

## Data Availability

Data are contained within the article.
